# Old Stars and New Players in the Brain Tumor Microenvironment

**DOI:** 10.3389/fncel.2021.709917

**Published:** 2021-10-06

**Authors:** Elena Parmigiani, Marta Scalera, Elisabetta Mori, Elena Tantillo, Eleonora Vannini

**Affiliations:** ^1^Embryology and Stem Cell Biology, Department of Biomedicine, University of Basel, Basel, Switzerland; ^2^Neuroscience Institute, Consiglio Nazionale delle Ricerche (CNR), Pisa, Italy; ^3^BIO@SNS Laboratory, Scuola Normale Superiore, Pisa, Italy

**Keywords:** glioblastoma, glioma, peritumoral tissue, neural activity, glial cells, tumor-associated microglia/macrophages (TAM), reactive astrocyte, OPCs

## Abstract

In recent years, the direct interaction between cancer cells and tumor microenvironment (TME) has emerged as a crucial regulator of tumor growth and a promising therapeutic target. The TME, including the surrounding peritumoral regions, is dynamically modified during tumor progression and in response to therapies. However, the mechanisms regulating the crosstalk between malignant and non-malignant cells are still poorly understood, especially in the case of glioma, an aggressive form of brain tumor. The presence of unique brain-resident cell types, namely neurons and glial cells, and an exceptionally immunosuppressive microenvironment pose additional important challenges to the development of effective treatments targeting the TME. In this review, we provide an overview on the direct and indirect interplay between glioma and neuronal and glial cells, introducing new players and mechanisms that still deserve further investigation. We will focus on the effects of neural activity and glial response in controlling glioma cell behavior and discuss the potential of exploiting these cellular interactions to develop new therapeutic approaches with the aim to preserve proper brain functionality.

## Introduction

Gliomas are the most common primary brain tumors in adults and children. They account for almost 30% of all primary brain tumors and 80% of malignant brain tumors (Weller et al., 2015). Although new therapeutic strategies are continuously under investigation, gliomas remain associated with high morbidity and mortality, especially in the case of glioblastoma multiforme (GBM), the most aggressive form of glioma (Patel et al., 2019; Liang et al., 2020). Hence, treatment of gliomas represents one of the hardest challenges of our times for neuro-oncologists. The standard of care for GBM patients consists in a maximal safe surgical resection followed by cycles of radio- and chemotherapy. However, this therapeutic regimen gives only partial benefits to the patients. Complete glioma eradication is usually prevented by the invasive nature of these tumors and by recurrence due to therapy-resistance (Bahadur et al., 2019). In addition, GBM patients also suffer from devastating neurological deterioration induced by the growing tumor mass as well as the aggressive treatments. In most of the cases, neurodegeneration and other tumor-associated secondary effects contribute to an unfavorable prognosis in glioma patients (Gibson and Monje, 2012; Zetterling et al., 2020).

For a long time, cancer research has mainly focused on understanding the biology of glioma cells and investigating the aberrant pathways that guide tumor onset and progression. The concept that the interaction between glioma cells and the tumor microenvironment (TME) is crucial in driving tumor growth has emerged lately (Hanahan and Coussens, 2012). Understanding microenvironmental determinants that contribute to glioma progression is therefore a major focus of current research, with the aim of discovering new therapeutic targets and strategies (Johung and Monje, 2017).

In order to sustain their growth, glioma cells co-opt brain-resident cells by establishing different communication routes which include secreted molecules, gap junctions, tunneling nanotubes, and extracellular vesicles (Osswald et al., 2015; D’Alessio et al., 2019; Jung et al., 2019; Lane et al., 2019; Matarredona and Pastor, 2019; Gao et al., 2020). Some of these mechanisms have been recently unraveled and exploited to develop new therapeutic strategies. However, the complex cell-cell interactions within the glioma TME are still largely unknown. The TME is also dynamically shaped during disease progression and eventually adapts in response to therapies (Venkatesh et al., 2015; D’Alessio et al., 2019).

Neurons and glial cells (microglia, astrocytes, and oligodendrocytes) not only cooperate to maintain brain homeostasis, but also represent major TME components that substantially contribute to tumor growth. Although the role of microglia has been extensively investigated over the last decade, some important questions still remain open. In contrast, the contribution of oligodendrocytes and, to some extent, astrocytes have been poorly addressed. Important advances have been made in the study of neuronal regulation of glioma development, but some aspects remain controversial. In addition, secondary effects due to glioma growth, like epilepsy and edema, can profoundly affect neuron functionality and lead to cognitive deficits through mechanisms that are not completely known. Therefore, a better understanding of the cellular and molecular dynamics within the TME is urgently needed in order to efficiently counteract glioma progression and improve patients’ survival and quality of life.

## Tumor-Associated Microglia and Macrophages: A Leading Player in the TME

Microglia cells are the main immune cell population in the brain (Korin et al., 2017). By continuously scanning the central nervous system (CNS) tissue, they act as “sentinels” that react to any type of pathogens or insult, and are implicated in virtually all CNS pathologies (Hanisch and Kettenmann, 2007). In the glioma field, microglial cells are commonly grouped together with infiltrating monocyte-derived macrophages (MDMs). Microglia and MDMs are collectively named as tumor-associated macrophages (TAMs). Accounting for up to 50% of the tumor mass, TAMs are a central component of the TME, and they play essential roles during tumor progression, recurrence and response to therapy (Gutmann and Kettenmann, 2019). For these reasons, TAM-targeting therapies have recently gained attention in the field as a promising way to treat gliomas (Kowal et al., 2019).

Microglia and MDMs are phenotypically similar and share the expression of markers such as Iba1, CD11b, F4/80, MerTK, and CSF-1R (Greter et al., 2015). Traditionally, their discrimination has been based on the different expression levels of CD45: while microglia are CD11b^+^CD45^low^-expressing cells, MDMs express high levels of CD45 (CD11b^+^CD45^high^). Thanks to gene expression profiling studies, more specific markers have been recently identified and are now widely used. These include P2RY12 (Butovsky et al., 2014), Sall1 (Buttgereit et al., 2016), Tmem119 (Bennett et al., 2016), and Hexb (Masuda et al., 2020) that are exclusively expressed by brain-resident microglia cells, while CD49d (Bowman et al., 2016) or Cxcr4 (Werner et al., 2020) are preferentially expressed by peripheral MDMs. However, activated microglial cells acquire some morphological and phenotypical characteristics of MDMs, making the discrimination of these two myeloid cell types problematic (Greter et al., 2015). Identifying the brain-resident vs. peripheral TAMs is important in light of functional differences that have been recently revealed and that possibly arise from a distinct ontogeny. Despite a common origin of microglia and MDMs in the yolk sac, MDMs derive from subsequent wave(s) of progenitors during development and are constantly replenished by bone marrow-derived monocytes in some adult tissues, while in others they maintain themselves locally (Ginhoux and Guilliams, 2016). In contrast, microglia cells originate from unique progenitors and colonize the brain early on during development. They remain in the brain tissue throughout life and self-maintain through a fine balance of proliferation and apoptosis with no need of contribution from the periphery, at least in physiological conditions (Ginhoux et al., 2010; Askew et al., 2017; Tay et al., 2017). In pathological conditions such as brain tumors, the blood brain barrier is compromised and a significant number of MDMs can colonize the brain and participate in disease evolution. Interestingly, when transplanted into a healthy brain, MDMs acquire some microglial-like traits under the influence of the host microenvironment, but they also maintain specific ontogeny-driven features (Bruttger et al., 2015; Bennett et al., 2018). A similar phenomenon has been observed in gliomas, where common and ontogeny-specific programs cooperate to educate microglia and MDMs to support tumor growth (Bowman et al., 2016). For instance, in the TME microglia usually show higher expression of pro-inflammatory genes (Bowman et al., 2016). Conversely, the expression of antigen processing and presentation molecules and of immunosuppressive cytokines is enriched in MDMs and, therefore, they might be more effective in hampering a proper T cell-mediated anti-tumor response (Bowman et al., 2016; Klemm et al., 2020). Differences in the expression profile of brain-resident microglia and peripheral MDMs have been recently confirmed also in humans, where they even vary depending on the brain tumor type. While the TME of less aggressive IDH mutant gliomas is dominated by microglia, in high grade gliomas the contribution of peripheral MDMs is higher. Moreover, TAMs with an immunosuppressive phenotype are particularly abundant in brain metastasis compared to primary gliomas (Friebel et al., 2020; Guldner et al., 2020; Klemm et al., 2020). Finally, a specific MDMs-related gene signature correlates with poor survival of glioma patients (Bowman et al., 2016; Friebel et al., 2020; Klemm et al., 2020). Different transcriptional responses have also been observed in microglia and MDMs after radiotherapy (Akkari et al., 2020) or anti-CD47 immunomodulatory treatment (Hutter et al., 2019).

Microglia activation in response to brain tumors has been extensively studied in mouse models of GBM (Bowman et al., 2016; Gutmann and Kettenmann, 2019; Friebel et al., 2020; Klemm et al., 2020) and in glioma patients (Müller et al., 2017; Sankowski et al., 2019; Friebel et al., 2020; Klemm et al., 2020). However, it remains unclear whether TAMs initially attempt to control tumor growth before being reprogrammed by tumor cells. Some studies have suggested that microglia and macrophages from healthy individuals may have the ability to delay the proliferation of brain tumor initiating cells *in vitro* (Sarkar et al., 2014), although these data still need to be confirmed. What is certainly known is that shortly after encountering tumor cells, TAMs are “educated” to promote and sustain tumor growth and invasion (Hambardzumyan et al., 2015). Multiple mechanisms inducing TAMs education have been described and these include tumor-released cytokines (Komohara et al., 2008; Zhou et al., 2015; Kloepper et al., 2016; De Boeck et al., 2020), metabolites (Colegio et al., 2014; Shen et al., 2016; Takenaka et al., 2019) and extracellular vesicles (Maas et al., 2020), or cell-cell contact between TAMs and malignant cells (Chia et al., 2018). Tumor cells can also produce chemokines like CCL2, CCL5, CX3CL1, and CSF1 to attract microglia and MDMs (Gutmann and Kettenmann, 2019).

Once activated, TAMs are reprogrammed to produce factors that stimulate glioma cell proliferation (Coniglio et al., 2012; Hammond et al., 2019), invasion (Markovic et al., 2009; Hu et al., 2014), and therapy resistance (Quail et al., 2016). For example, TAMs favor glioma invasion by remodeling the extracellular matrix (ECM). Factors released by both TAMs and tumor cells induce the reciprocal expression of MMP2, MMP9, and MT1-MMP, that degrade the ECM facilitating glioma migration (Wick et al., 2001; Markovic et al., 2009; Hu et al., 2014). Interestingly, MMP2 is released as an inactive molecule and requires the presence of MT1-MMP on the surface of TAMs to be activated (Markovic et al., 2009), indicating that TAMs and glioma cells can coordinate their functions in the TME. In addition, TAMs can indirectly promote glioma progression by favoring angiogenesis (De Palma et al., 2008) and immunosuppression. *In vitro* studies demonstrated that conditioned medium from glioma cells was sufficient to inhibit the anti-cancer properties of TAMs, including phagocytosis and T-cell stimulation (Wu et al., 2010). TAMs isolated from human glioma specimens showed an impaired ability to activate anti-tumor T cells (Hussain et al., 2006), possibly due to an unbalanced expression of T cell stimulatory vs. inhibitory molecules (Klemm et al., 2020). Therefore, upon activation, TAMs acquire a unique multifunctional activation state (Hambardzumyan et al., 2015) which has been recently confirmed and expanded by single cell RNA-sequencing (scRNA-seq) studies. A complex scenario has emerged describing an heterogeneous population of TAMs which comprises multiple activation states and distinct subpopulations characterized by specific expression profiles (Müller et al., 2017; Sankowski et al., 2019; Friebel et al., 2020; Guldner et al., 2020; Klemm et al., 2020; Pombo Antunes et al., 2021). This might be the consequence of an intrinsic heterogeneity and plasticity of CNS myeloid cells in response to different stimuli (Masuda et al., 2020) and/or it might reflect spatial heterogeneity within the TME. For example, TAMs expressing pro-inflammatory and immunostimulatory genes co-exist with anti-inflammatory TAMs populations (Guldner et al., 2020; Klemm et al., 2020). However, pro-inflammatory cytokines genes, like IL1, are more expressed by TAMs at the tumor periphery compared to the tumor core where, instead, anti-inflammatory genes like TGFβ are upregulated (Darmanis et al., 2017). Within the tumor core, a subpopulation of CD206^+^CD169^+^CD209^+^ macrophages was found specifically associated with blood vessels (Friebel et al., 2020) and TAMs expressing hypoxic and pro-angiogenic genes HIF1A and VEGF-A have been described in human gliomas and might be enriched in hypoxic regions (Darmanis et al., 2017; Sankowski et al., 2019). However, a more precise functional characterization of those TAM subpopulations is largely missing. In contrast to other neurodegenerative and inflammatory diseases, for which sequential microglia activation states have been described (Keren-Shaul et al., 2017; Mathys et al., 2017; Sousa et al., 2018; Hammond et al., 2019; Masuda et al., 2019), the temporal dynamics of TAMs education in brain tumors are still largely unknown. In addition, the functional differences among distinct TAM subpopulations and their clinical relevance is still poorly defined.

Interestingly, specific mutations in tumor cells can indirectly shape the immune TME and, in particular, TAMs recruitment and activation. NF1 or PTEN deletion in glioma cells increased microglia and macrophages infiltration *in vitro* and in mouse models (Wang et al., 2017; Chen et al., 2019). AKT1 overexpression also enhances physical interaction of microglia with AKT1^+^-tumor cells in a zebrafish model of brain tumors (Chia et al., 2019). In contrast, IDH mutation is associated with diminished production of chemoattractant cytokines and consequent lower numbers of TAMs in mouse models, and with a less immunosuppressive TAM phenotype in humans (Amankulor et al., 2017; Friebel et al., 2020; Klemm et al., 2020). This evidence highlights the ability of microglia and MDMs to respond and adapt to the TME and emphasize the complexity and heterogeneity of TAMs.

### TAM Re-education as a Therapeutic Strategy to Cure Gliomas

Given their central role in tumor progression, TAMs represent a promising therapeutic target. Over the last years, multiple approaches have been tested in preclinical and clinical studies with the goal of re-educating TAMs to exploit their anti-tumorigenic potential (Kowal et al., 2019). These strategies include the use of inhibitors of myeloid-specific receptors like CSF-1R (Pyonteck et al., 2013) and MARCO (Georgoudaki et al., 2016), molecules targeting key factors involved in TAM polarization (Kaneda et al., 2016), or agents that boost TAMs phagocytic activity like anti-CD47 antibodies (Hutter et al., 2019). However, despite promising results in different cancer types (Mantovani et al., 2017), these approaches have failed to significantly improve the overall survival of GBM patients compared to traditional treatments (Butowski et al., 2016). Indeed, after an initial phase of tumor regression, recurrence occurred upon CSF-1R inhibitor treatment in preclinical mouse models of glioma (Quail et al., 2016). Interestingly, therapy-resistance is induced by tumor-derived factors that activate pro-survival pathways in TAMs (Quail et al., 2016), suggesting a continuous and dynamic TAMs-tumor cells crosstalk during tumor evolution in response to therapy. Moreover, a population of Lgals3^+^-myeloid cells resistant to CSF-1R inhibitors was recently identified in physiological conditions (Zhan et al., 2020) and also observed in mouse models of GBM (Ochocka et al., 2021). However, additional investigations are needed to elucidate the mechanisms driving therapy-resistance and develop approaches that increase the efficiency of TAM re-educating agents. Promising results come from combinatorial strategies where TAM immunomodulatory molecules significantly enhanced the efficacy of other treatments (Quail et al., 2016; Kowal et al., 2019; Akkari et al., 2020). Also, some non-cancer related FDA-approved drugs have demonstrated an unexpected ability to stimulate a TAM anti-tumor activity and might be repurposed as adjunctive treatments: examples are the antifungal Amphotericin B (Sarkar et al., 2014), the antibiotic thiostrepton (Hu et al., 2021), the iron supplement ferumoxytol (Zanganeh et al., 2016), and the vitamin B3 (Sarkar et al., 2020). Interestingly, TAM re-educating strategies were also effective at reducing astrocytes reactivity, oligodendrocytes loss and at ameliorating the neurological deficits associated with chemotherapy (Gibson et al., 2019).

Although the main line of evidence points toward a pro-tumorigenic role of TAMs and prompts the implementation of TAM re-educating therapies, there are some important exceptions that need to be considered. Maximov et al. (2019) described a surprising anti-tumorigenic role of TAMs, and in particular CCR2^+^ MDMs, in SHH-medulloblastoma, where TAMs deletion significantly accelerated tumor growth. These findings could be explained by the higher phagocytic activity of cerebellar microglia compared to other brain areas and potentially reflect regional-differences (Ayata et al., 2018). But they also suggest caution when designing TAM re-educating strategies for brain tumors.

## Another Protagonist of the TME: Reactive Astrocytes

Astrocytes are the most abundant glial cells in the CNS. One of the main characteristics of these cells is their ability to respond to multiple types of injuries and to participate in different diseases (Buffo et al., 2010). It is therefore not surprising that they can react also to the presence of tumor cells. In a similar way to microglia, astrocytes are efficiently co-opted by tumor cells to sustain their proliferation, survival, migration and therapy resistance (Wasilewski et al., 2017; Brandao et al., 2019). GBM patients with high expression of reactive astrocytes genes show a worse prognosis and tumor cells co-transplanted with astrocytes develop more aggressive tumors in mice (Mega et al., 2020). Moreover, astrocytes can be a cell-of-origin of gliomas, as they give rise to high-grade brain tumors upon transformation (Bachoo et al., 2002; Zhu et al., 2005). Interestingly, different mutations can impose an astrocytic vs. oligodendrocytic fate to tumor cells, with the first usually associated with more aggressive tumors. For example, cells with aberrant EGFR and Ink4A/Arf deletion acquire expression of astrocytes markers (Bachoo et al., 2002), while PDGFB overexpression is sufficient to de-differentiate astrocyte cultures and to form oligodendroglioma or oligoastrocytoma *in vivo* (Dai et al., 2001). These findings are consistent with the differences described in human glioma subtypes, where an astrocytic gene signature is enriched in the classical subtypes characterized by EGFR amplification and Ink/Arf deletion (Verhaak et al., 2010). Also the contribution of different astrocyte subpopulations to the TME can vary among GBM subtypes (John Lin et al., 2017). Interestingly, a gene signature defining astrocytes activation upon co-culture with GBM cells was significantly associated with mesenchymal GBM (Mega et al., 2020), suggesting a prominent role of non-malignant reactive astrocytes in this tumor subtype.

Astrocytes are characterized by broad morphological and functional heterogeneity that may be the consequence of regional and ontogenetic differences (Khakh and Deneen, 2019). Astrocyte heterogeneity could also affect the tumorigenic potential of these cells, although the mechanisms behind are yet to be unraveled. It has been shown that GFAP^+^ and GLAST^+^ astrocytes, when transformed, give rise to GBM with different features: GFAP^+^ tumor cells are intrinsically more proliferative than GLAST-derived tumors that, instead, may rely more on the TME, as suggested by enhanced recruitment of non-malignant glial cells (Irvin et al., 2017). Marker combinations can be used to subdivide astrocytes in discrete subpopulations that co-exist in healthy brains and can give rise to aggressive gliomas. These astrocytes subpopulations and their malignant analogs display different molecular and functional properties (John Lin et al., 2017).

Whether such heterogeneity characterizes also non-malignant astrocytes in TME remains undefined. By comparing gene expression profiles of TME-associated astrocytes in low and high grade gliomas, Katz et al. (2012) identified a subpopulation of astrocytes specifically localized in perivascular regions of aggressive tumors, where also a population of CD44^+^ stem-like glioma cells reside. These astrocytes express high levels of osteopontin (or SPP1), a CD44 ligand that induce stemness in glioma cells (Pietras et al., 2014). Both osteopontin and CD44 correlate with a poor overall survival in patients, suggesting a role of perivascular-niche astrocytes in supporting glioma stem cells. Astrocytes expressing a phosphorylated form of PDGFRβ and associated with blood vessels favor brain metastases dissemination and can be targeted to impair metastatic growth (Gril et al., 2013). Another subpopulation of astrocytes that displays high levels of the immune checkpoint PD-L1 and activation of the immunomodulatory factor STAT3 has also been observed in the peritumoral area, where it might constitute a barrier against anti-tumor T lymphocytes (Priego et al., 2018; Henrik Heiland et al., 2019).

Peritumoral regions are implicated in tumor invasion and multiple studies have demonstrated a role of astrocytes in promoting tumor migration in different ways: by expressing the chemoattractant factor GDNF (Shabtay-Orbach et al., 2015), by releasing MMPs (Le et al., 2003) or by inducing MMPs upregulation in tumor cells (Chen W. et al., 2016). Similar to TAMs, astrocytes can cooperate with tumor cells in remodeling the ECM *via* a mechanism involving plasminogen activation. Plasminogen is produced by glioma cells and converted to plasmin by the protease uPA, which is activated by the uPA receptor expressed on astrocytes. Plasmin mediates MMP2 activation and ECM degradation favoring tumor invasion (Le et al., 2003). Nonetheless, in different contexts, such as the initial phase of brain metastasis, astrocyte-derived PA and plasmin can be cytotoxic for tumor cells that enter in a naïve brain parenchyma. To overcome this effect, tumor cells upregulate the PA inhibitors serpins allowing brain infiltration and metastasis dissemination (Valiente et al., 2014). Hence, although in the early phase of metastasis astrocytes seem to counteract brain invasion, later on they are converted by metastatic cells to promote tumor growth, similarly to what they do in primary glioma (Wasilewski et al., 2017). The events triggering the pro-tumorigenic education of astrocytes in primary and metastatic brain tumors are partially unknown. One described mechanism involves the establishment of direct cell-cell contacts to transfer signaling molecules from tumor cells to astrocytes. These molecules induce astrocytes to release cytokines which, in turn, promote tumor growth (Chen Q. et al., 2016). Astrocytomas, but not oligodendrogliomas, exploit similar gap junctions-mediated connections to form functional cellular networks of tumor cells and rapidly exchanged second messengers and propagate calcium signaling (Osswald et al., 2015). Using similar intercellular communication, astrocytes can also protect metastatic tumor cells from apoptosis by sequestering calcium accumulated in tumor cells upon chemotherapy treatment (Lin et al., 2010).

Immunomodulation is another critical aspect of astrocyte reactivity (Linnerbauer and Rothhammer, 2020). Astrocytes can regulate the immune TME by responding and releasing a broad spectrum of cytokines and by communicating with multiple immune cell types (Priego and Valiente, 2019). Astrocytes expressing high levels of immunosuppressive cytokines, such as TGFβ and IL10, have been recently identified in human GBM samples (Henrik Heiland et al., 2019). JAK/STAT and STAT3 inhibition efficiently reverts astrocytes-related immunosuppression and delay tumor growth, indicating a central role of this pathway in controlling astrocytes immunomodulatory properties (Priego et al., 2018; Brandao et al., 2019; Henrik Heiland et al., 2019). Intriguingly, the presence of phosphorylated-STAT3^+^ immunosuppressive astrocytes is promoted by tumor and microglia cells. Reactive astrocytes can also induce the upregulation of immunosuppressive and tumor-promoting molecules in microglia and macrophages, establishing a positive feedback loop between TAMs and astrocytes in the TME (Priego et al., 2018; Henrik Heiland et al., 2019). Hence, the bidirectional crosstalk between astrocytes and microglia is crucial to define the immunosuppressive microenvironment in brain tumors (Matias et al., 2018). Both cell types, once activated, release cytokines that can boost each other’s activation. Importantly, distinct types of cytokines differently influence the activation states and, consequently, the secretome of astrocytes and microglia (Liddelow et al., 2017). Therefore, the microglia-astrocytes interconnection could be a promising therapeutic target that certainly deserves further studies.

Astrocytes have a major role in inducing brain tumor resistance to therapy. Radiation, for instance, can stimulate the release of multiple factors by reactive astrocytes. These factors can support glioma stemness and promote tumor recurrence (Berg et al., 2021). Astrocytes collaborate with microglia to clear oncolytic viral particles, leading to severe implications for the therapeutic use of oncolytic viruses in gliomas (Kober et al., 2015). Also, it remains to be addressed whether astrocytes affect immunotherapy responses by virtue of their immunomodulatory properties and expression of immune checkpoint molecules (Priego et al., 2018; Henrik Heiland et al., 2019; Priego and Valiente, 2019).

Altogether, it is clear that astrocytes actively shape the TME of gliomas and metastatic brain tumors, playing mainly a tumor-promoting role. In the recent years some, although limited in number, innovative approaches targeting astrocytes in brain tumors have been tested in preclinical and clinical trials (Wasilewski et al., 2017). The JAK/STAT pathway is one of the best candidates, being at the crossroad of multiple astrocytes-secreted cytokines, like IL10 and IL6, as well as a critical regulator of astrocytes immune response. Legasil^®^, a nutraceutical product containing a natural polyphenolic flavonoid called Silibinin, inhibits activation of STAT3 in reactive astrocytes and is able to reduce brain metastasis and significantly increase overall survival rate (Priego et al., 2018). Although tested in a small cohort of patients only with metastatic brain tumors, the results are certainly promising. Interfering with intercellular gap junctions is another way to limit tumor-astrocytes communication. Carbenoloxone, a pan-connexin inhibitor, efficiently blocks intercellular calcium waves propagation among astrocytoma cells (Osswald et al., 2015). Beyond its anti-inflammatory function, meclofenamate was found to inhibit also Cx43 gap junctions (Harks et al., 2001) and is now under investigation in a clinical trial for brain metastatic patients. Moreover, astrocytes heterogeneity and astrocytes-specific factors might also be exploited as novel diagnostic strategies. For example, Katz et al. (2012) defined a tumor-associated astrocytes gene signature which efficiently predicts the survival of patients specifically with proneural GBM subtype. However, many biological and molecular aspects of TME-associated astrocytes still need to be untangled in order to fully exploit their therapeutic potential.

## The Role of Neural Stem and Progenitor Cells

Neural stem cells (NSCs) and progenitors of the subventricular zone have been proposed as a cell-of-origin of gliomas (Alcantara Llaguno et al., 2009; Lee et al., 2018). Notably, differentiation of NSCs toward the neuronal lineage represents a hurdle for the initiation of brain tumors. Immature and mature neurons are resistant to transformation after loss of tumor suppressor genes, a condition that rapidly triggers tumor formation in neural progenitors and glial cells (Alcantara Llaguno et al., 2019).

In addition to being a cell-of-origin of tumors, neural progenitors can contribute to shape the TME. Non-malignant neural progenitor cells are strongly attracted by tumor cells and preferentially accumulate at the tumor border. Differently from the majority of cells composing the TME, their presence delays tumor growth, as demonstrated by enhanced mice survival when GBM cells are co-transplanted with neural precursors (Glass et al., 2005). Mechanistically, neural progenitors trigger cell death by secreting endovanilloids which stimulate the vanilloid receptor TRPV1, specifically expressed by mouse and human GBM cells (Stock et al., 2012). However, glioma cells might have developed a way to counteract the anti-tumorigenic effect of neural progenitors. In a recent study on mouse NCSs, Lawlor et al. (2020) discovered that, when in direct contact, malignant-NSCs induce quiescence in wild-type (wt)-NSCs *via* NOTCH signaling activation. In this way, they rapidly outcompete wt-NSCs in numbers. A crosstalk between tumor cells and neural progenitors remains to be confirmed in humans, but it might be particularly relevant in young patients, where progenitor cells are still present. However, it should be considered that in inflammatory contexts NSCs can interact with microglia/macrophages inducing immunosuppression (Peruzzotti-Jametti et al., 2018). Given their high tropism for tumors and their tumor-suppressive function, neural progenitors could be exploited as an alternative therapeutic strategy to counteract gliomas.

## Oligodendrocytes and Oligodendrocyte Progenitor Cells: An Underestimated Player in the TME

Using multiple transgenic mouse models, different groups have demonstrated that oligodendrocyte progenitor cells (OPCs) can be a cell-of-origin of gliomas (Liu et al., 2011; Sugiarto et al., 2011; Galvao et al., 2014). The presence of oligodendrocytes with the typical “fried egg-shape” has been historically the main histopathological criteria to define human oligodendroglioma (Gupta et al., 2005). More recently, comprehensive molecular genetic profiling and scRNA-sequencing studies have confirmed the presence of OPC-like glioma cells not only in oligodendroglioma but also in other glioma entities, like astrocytoma and GBM, although with varying abundance (Venteicher et al., 2017; Neftel et al., 2019). Of note, the proneural GBM subtype is characterized by alteration of *PDGFRA* and high expression of oligodendrocytic markers (Verhaak et al., 2010; Wang et al., 2017).

The function of OPCs and oligodendrocytes within the TME has been neglected for a long time. A recent bioinformatic method that predicts tumor-TME interactions from scRNA-seq datasets revealed a significant infiltration of non-malignant oligodendrocytes in the TME of proneural GBM compared to other GBM subtypes (Caruso et al., 2020). The authors also identified ligand-receptor pairs that suggest crosstalk between malignant and non-malignant oligodendrocytes. For example, infiltrating oligodendrocytes express high levels of PDGFA that, *via* activation of its receptor PDGFRα on glioma cells, could fuel tumor proliferation (Cao, 2013; Caruso et al., 2020). Other evidence, mainly *in vitro*, suggests the existence of a bidirectional communication between oligodendrocytes and tumor cells within the TME. Conditioned media from human glioma cell lines increases the survival of OPCs. In turn, OPC-derived factors like EGF and FGF1, promote stemness and chemotherapy resistance of glioma cells (Hide et al., 2018). In contrast, a negative correlation between Wif-1^+^ non-malignant oligodendrocytes and the proliferation index of human astrocytoma cells has been reported, suggesting an anti-tumorigenic role of oligodendrocytes *via* Wnt inhibition (Asslaber et al., 2017). Although the function of non-malignant OPCs needs further investigation, the accumulation of these cells at the tumor border of human gliomas suggests that they might play a strategic role within the TME (Asslaber et al., 2017; Hide et al., 2018). In the tumor periphery TAMs are also particularly abundant suggesting a TAMs-OPCs crosstalk. Indeed, it has been shown that macrophage-derived soluble factors sustain OPCs viability (Hide et al., 2018). In addition, Huang and colleagues described a population of glioma-associated OPCs that promotes neovascularization and facilitates tumor growth (Huang et al., 2014). These PDGFRα^+^ OPCs are recruited at the tumor border through PDGFC released by TME cells, including TAMs. Interestingly, mice with either *PDGFRA* or *PDGFC* deletion display a normalized vascular network and a significant decrease in tumor volume. OPCs could also influence microglia *via* TGFβ, which is essential for proper microglia development and maintenance. Pharmacological and genetic depletion of NG2^+^ OPCs indirectly causes downregulation of TGFβ signaling in microglia with consequent alteration of microglia homeostasis. Although in the absence of a pathological condition no changes of activation markers were detected in microglia (Liu and Aguzzi, 2020), it would be interesting to investigate the effects of OPCs depletion on microglia in the context of a tumor. IGF-1 is, instead, crucial for OPCs survival and myelination during brain development, when it is mainly produced by CD11c^+^ microglia cells (Wlodarczyk et al., 2017), a subset of microglia also identified in human gliomas (Friebel et al., 2020). IGF-1, released by TAMs, promotes brain tumor progression and recurrence in response to prolonged administration of CSF-1R inhibitors (Quail et al., 2016; Yao et al., 2020) and might have also some still unexplored effects on OPCs/oligodendrocytes of the TME.

The crosstalk between glioma cells and oligodendrocytes is demonstrated also by the observation that tumor cells use white matter tracts to disseminate into the brain and escape radiotherapy, precluding a complete tumor eradication. Despite being a well-known characteristic of aggressive gliomas (Cuddapah et al., 2014), the mechanisms behind this phenomenon are unclear. Wang et al. (2019) proposed a NOTCH-SOX2-SOX9 axis driving tropism of glioma cells for white matter tracts. They found that CD133^+^ bona-fide glioma stem cells are preferentially associated with portions of nerve fibers, where myelin is lost and unconstrained axonal signals stimulate glioma cell survival. Partial demyelination nearby white matter infiltration was also recently reported by Brooks et al. (2021). The authors described, in addition, axonal degeneration, OPCs recruitment and microglia activation: all events usually occurring in demyelinated lesions (Franklin and Ffrench-Constant, 2008). Surprisingly, this white matter injured-like microenvironment is sufficient to stop proliferation of infiltrating tumor cells and to upregulated SOX10 which induces differentiation of tumor cells toward the oligodendrocyte lineage (Brooks et al., 2021). Therefore, in white matter tracts tumor cells may encounter opposite signals stimulating stemness or differentiation. What determines the final outcome is still unclear. More importantly, these reports revealed two intriguing aspects: (i) white matter tracts may play an active role in shaping the fate of tumor cells and not just be a route for tumor cell invasion; (ii) myelinating oligodendrocytes directly interact with tumor cells. Mechanism triggering myelin damage as well as signals regulating OPCs/oligodendrocytes-glioma communication need to be clarified with further investigation.

In addition, it is important to remember that neuronal electrical activity not only promotes tumor growth, as we will describe later (Venkatesh et al., 2015, 2017), but can also stimulate OPCs proliferation and restore myelination in demyelinated lesions (Gibson et al., 2014; Ortiz et al., 2019). Notably, OPCs express high levels of NLGN3 which, together with neuronal neurexin, forms axo-glial contacts that favor OPCs proliferation and myelination (Proctor et al., 2015). Activity-dependent release of NLGN3 by OPCs has been observed, but the molecular mechanism is still unknown and doesn’t involve ADAM10 cleavage, as in the case of neuronal-derived NLGN3 (Venkatesh et al., 2017). However, more studies are required to clarify the role of OPC-derived NLGN3 in the TME. OPCs possess also additional characteristics which might be relevant during tumor evolution, like the formation of functional synapses with neurons, the establishment of cellular networks that propagate calcium signaling and the ability to buffer the potassium released after neuronal activity (Menichella et al., 2006; Bergles et al., 2010; Parys et al., 2010; Venkatesh et al., 2019). All these features might allow non-malignant OPCs to functionally integrate into tumor cell networks and tune depolarizing currents in glioma cells, modulating their proliferation (Osswald et al., 2015; Venkatesh et al., 2019). Additional studies will help to understand a possible neuron-OPC-glioma interplay that could drive tumor progression.

The fact that oligodendrocytes possess immunomodulatory properties is a relative recent concept that is still poorly investigated in gliomas, but that might have important implications for disease progression and therapy response. Data from other CNS diseases have recently proven the ability of OPCs to directly interact with immune cells and to modulate inflammation. In a mouse model of preterm white matter injury, OPCs and particularly immature oligodendrocytes are capable of responding to neuroinflammation by upregulating cytokines and the innate immune response receptor TLR3. OPCs/oligodendrocytes stimulated *in vitro* with a natural ligand of TLR3 secrete factors that activate an inflammatory and phagocytic response in microglia (Boccazzi et al., 2021). OPCs can also upregulate MHC-I/II in response to IFNγ and are able to cross-present antigens and stimulate T cells (Falcão et al., 2018; Kirby et al., 2019). In multiple sclerosis plaques, this ability leads to presentation of myelin antigens and a consequent increase of inflammation, OPCs death and impaired remyelination (Kirby et al., 2019). However, in contexts where a T cell response is required to kill tumor cells, the ability of OPCs to activate cytotoxic T cells might be helpful to counteract tumor growth. The immunomodulatory activity of OPCs/oligodendrocytes can also be exerted through recruitment of innate immune cells. However, OPCs can also impair immune cells function and limit inflammation by upregulating factors, like Sema3A (Majed et al., 2006) or FasL (Dowling et al., 1996), inducing immune cells apoptosis. In addition, microglia phagocytosis and clearance can be inhibited by an excess of myelin debris (Shen et al., 2021) which can be a consequence of tumor infiltration along white matter tracts.

Finally, cell adhesion and ECM molecules, as well as MMPs and metalloproteases inhibitors, have been found enriched in OPCs *in vitro* and *in vivo* (Parolisi and Boda, 2018), indicating that these glial cells are an additional and crucial player in tissue remodeling. Moreover, oligodendrocytes release of MMP9 upon IL1β stimulation or after white matter injury, facilitates endothelial tube formation and angiogenesis (Pham et al., 2012), further suggesting a potential implication of oligodendroglia in neovascular remodeling within the TME.

Therefore, although increasing evidence propose OPCs and oligodendrocytes as potential key players in the TME, more work is required to elucidate their roles during disease progression.

## The Multifaceted Role of Neuronal Cells in Glioma

Recent important findings unravel an active role of neurons within the TME. However, complex relationships between multiple types of neurons make this mechanism difficult to disentangle (Deisseroth et al., 2004; Ge et al., 2007). During normal development, electrical activity influences the formation of central and peripheral neural system (Gafarov, 2018). In particular, CNS maturation is shaped by patterned waves of electrical activity inducing calcium transients (Wong et al., 1995; Corlew et al., 2004). In the adult brain, excitatory neurons modulate proliferation and differentiation of stem cells in the subgranular zone of the dentate gyrus and in the subventricular zone (Deisseroth et al., 2004; Paez-Gonzalez et al., 2014). Moreover, adult neurons regulate Schwann cell proliferation and survival in the PNS (Maurel and Salzer, 2000) and glial precursors proliferation and myelin plasticity in the CNS, conditioning brain structure and function (Scholz et al., 2009; Takeuchi et al., 2010). Similarly, neurons can also affect glioma proliferation (Johung and Monje, 2017; Tantillo et al., 2020b).

Recent studies have proven that neural activity affects glioma cells behavior in a region-specific manner. Indeed, visual deprivation only affects glioma proliferation when the tumor is located in the visual cortex, but not in the motor cortex (Tantillo et al., 2020b). In addition, the activation of different neuronal populations may have a differential action on glioma growth. In the latest years it has been demonstrated that, while pyramidal cell activity promotes proliferation in both murine and patient-derived high-grade gliomas (Venkatesh et al., 2015, 2017; Tantillo et al., 2020b), the selective stimulation of parvalbumin-positive interneurons elicits the opposite effect in glioma-bearing mice (Tantillo et al., 2020b). Similarly, in other types of cancer such as breast cancer, the stimulation of parasympathetic (cholinergic) afferents reduces tumor growth and the stimulation of sympathetic (noradrenergic) nerves accelerates it (Kamiya et al., 2019). Several studies suggest also that the release of secreted molecules such as neurotransmitters and neurotrophins exert a relevant role in the neural regulation of glioma proliferation. For instance, direct interaction between glioma cell lines and neurons leads to an upregulation of functional GABA A receptor in glioma cells, which strongly correlates with the malignancy of brain tumors (Synowitz et al., 2001). However, since both neurons and glioma cells can induce activity-dependent secretion, unraveling the cellular source of these fundamental factors is technically challenging and require further investigation.

Despite the latest evidence demonstrating a tumor-promoting effect of neurons, an anti-proliferative effect of peritumoral neurons on glioma cells has also been described (Liu et al., 2013). Peritumoral neurons are capable of restraining glioma progression through the pivotal role of PD-L1, whose signaling in brain cells is important for GBM patient survival (Liu et al., 2013). High expression of PD-L1 by peritumoral neurons positively correlates with GBM prognosis, demonstrating the existence of a PD-L1-dependent interaction between peritumoral neurons and tumor cells (Liu et al., 2013). On the contrary, its expression on GBM cells promotes PD-1 receptor activation in TAM and T cells, inhibiting tumor cells phagocytosis and killing (Gordon et al., 2017; Litak et al., 2019).

All these studies depict a complex scenario of bidirectional interactions between neuronal and glioma cells. This entangled crosstalk needs to be completely dissolved in order to achieve a clear understanding of its underlying mechanisms, which may pose the foundation for the development of more effective therapeutic approaches.

### The Complex Role of Neurotransmitters and Neurotrophins

It is well-known that glioma cells express functional neurotransmitter receptors, which could interfere with mitogenic pathways. Glutamate is one of the most abundant molecules of the CNS, but its extracellular levels must be maintained low, in order to prevent hyperexcitability and guarantee a normal brain function. Therefore, the scavenger role of astrocytes, through Na^+^-dependent glutamate transporters (i.e., EAAT1 or EAAT2), is of paramount relevance to maintain this equilibrium (Bergles and Jahr, 1997; Danbolt, 2001; Robert and Sontheimer, 2014). Similarly, glioma cells express high levels of EEAT1/2. When EEAT1/2 is absent or mislocalized glutamate uptake is compromised; this event causes high levels of glutamate in the extracellular space that leads to excitotoxicity (Ye et al., 1999; Buckingham and Robel, 2013; Robert and Sontheimer, 2014; Campbell et al., 2015).

Tumor cells can also extrude glutamate, predominantly through the cystine-glutamate antiporter (system xc-, SXC) that exchanges intracellular glutamate for extracellular cysteine, normally involved in the generation of antioxidant glutathione (de Groot and Sontheimer, 2011). The biochemical source of glutamate for glioma cells is not entirely known. Some studies suggest that glutamate may be generated from glutamine *via* a glutaminase reaction. Other works, instead, propose that glutamate could be accumulated due to other metabolic impairments, as the decreased conversion in α-ketoglutarate by GLUD2 enzyme (de Groot and Sontheimer, 2011; Franceschi et al., 2018). Glioma cells express both ionotropic and metabotropic glutamate receptors (de Groot and Sontheimer, 2011) and the increased extracellular glutamate levels in proximity of the tumor mass (Behrens et al., 2000) promote survival, growth and migration in part through the activation of AMPA receptors and, consequently, Rho-Akt pathway (Ishiuchi et al., 2007). The high amount of glutamate acts in a paracrine/autocrine manner on tumor cells, facilitating their spread into the brain parenchyma. This event results in excitotoxicity, which damages neurons located in the tumor vicinity. Moreover, gliomas are known to increase the neural excitability of peritumoral neurons (Venkatesh and Monje, 2017), even if the mechanism that links hyperactivity and cell proliferation has still not completely clarified.

In a normal brain, GABA is the most important inhibitory neurotransmitter of the CNS and controls stem cell proliferation in the subventricular zone and hippocampus, limiting the generation of new neuroblasts (Young and Bordey, 2009; Song et al., 2012; Giachino et al., 2014; Blanchart et al., 2017). It has been demonstrated that glioma cells express GABA receptors, whose function is still under debate. Studies on patient-derived glioma cultures show that the expression of functional GABA A receptors correlates more with low-grade gliomas and oligodendrogliomas than GBM (Labrakakis, 1998; Smits et al., 2012). On the contrary, in GBM GABA A receptors are downregulated to reduce GABA inhibition of tumor cell proliferation (Jung et al., 2019). At the same time, other *in vitro* studies on human GBM samples suggest the opposite (D’Urso et al., 2012). In a p16 Arf^–/–^ PDGFB murine glioma model an endogenous ionotropic signaling has been identified within tumor cells, demonstrating that glioma cells not only respond to GABA, but they can also release it in the TME, limiting cell proliferation of both murine and patient-derived GBM cells (Blanchart et al., 2017). It has been proposed that GABA could have a role in tumor progression, as suggested by *in vivo* experiments using GABA B agonist (muscimol) and GABA A antagonist (bicuculline). Intriguingly, the effect of GABA is more pronounced in glioma cells with stem-like properties, suggesting a possible strategy to maintain a pool of quiescent tumor initiating cells (Blanchart et al., 2017).

Neurotrophins are a family of secreted molecules that influence many aspects of brain physiology. During malignancies, it is not clear whether they exert a pro or anti-tumorigenic effect on glioma cells (Garofalo et al., 2015; Venkatesh et al., 2015). Their controversial role is still under debate and may depend on their multifaceted functions. For instance, it is known that in pediatric tumors, such as neuroblastoma or medulloblastoma, tumor cells express neurotrophin receptors and the associated clinical outcome is favorable (Donovan et al., 1993; Nakagawara et al., 1993; Eberhart et al., 2001). In adult low-grade gliomas TrkA and TrkB are highly expressed, while their presence in GBM is weak, suggesting that those receptors might be involved only in the less aggressive phase of the disease (Wadhwa et al., 2003). Some studies have shown that the activation of the TrkA receptor through NGF treatment has an anti-mitotic and pro-differentiating effect on C6 glioma cells (Rabin et al., 1998; Kimura et al., 2002). Conversely, on human GBM cell lines, NGF seems to improve tumor proliferation *via* Notch signaling (Park et al., 2018). In addition, a phase II clinical trial with administration of NGF to patients with childhood optic gliomas led to a statistically significant improvement in vision respect to placebo-treated patients (Falsini et al., 2016).

Also the role of BDNF in the regulation of glioma growth remains controversial. BDNF is a peptide secreted in an activity-dependent manner (Hong et al., 2008). *In vitro* studies demonstrate that BDNF, through its receptor TrkB, stimulates high grade glioma cells proliferation (Xiong et al., 2013) and that BDNF release increases with neural activity (Venkatesh et al., 2015). At the same time, it has also been shown that BDNF overexpression in the hypothalamus has immune-augmenting properties, provoking an increased anti-tumor immune response and reducing the activity of proteins that would normally confer resistance to chemotherapy (Radin and Patel, 2017). Moreover, glioma-bearing mice reared in an enriched environment (EE) showed enhanced levels of BDNF together with significantly reduced tumor growth, suggesting a pivotal role for this neurotrophin in glioma proliferation (Garofalo et al., 2015). BDNF infusion was also found to reduce TAMs infiltration and activation, and to dampen glioma migration *via* inhibition of RhoA through the truncated TrkB.T1 receptor (Garofalo et al., 2015). These data also emphasize the idea that lifestyles and physical activity can have a direct impact on the brain microenvironment (Garofalo et al., 2015; Tantillo et al., 2020a), counteracting glioma progression.

## Neuronal Alterations Induced by Glioma Growth

Emerging evidence suggests that glioma cells exert a strong influence on neurons and neuronal activity. For instance, deficits in neurocognitive functioning frequently occur in glioma patients, heavily affecting their quality of life (Aaronson et al., 2011; Seano et al., 2019). The tumor mass can hamper brain functionality not only by mechanical compression on brain structures but also by releasing excitotoxins which provoke neuronal death and perturb synaptic transmission (van Kessel et al., 2017).

Approximately 30–50% of patients with brain tumors manifest seizure as an initial symptom of disease progression (van Breemen et al., 2007), making tumor-associated epilepsy (TAE) among the most common hallmarks of comorbidity in glioma patients (Radin and Tsirka, 2020). Interestingly, the incidence of developing TAE is higher in low compared to high grade tumors (van Breemen et al., 2007; You G. et al., 2012), but the reasons of such difference are not known. TAE often manifests as focal seizures with secondary generalization and, despite its major clinical and social impact, the pathophysiological causes are poorly understood. In addition, due to the complex heterogeneity of perturbed mechanisms, this kind of epilepsy is often refractory to antiepileptic treatments (You G. et al., 2012; Cowie and Cunningham, 2014). An important aspect that determines the insurgence of seizures is the localization of the tumor mass in the brain. The proximity to the cortical gray matter is a fundamental factor: tumors that are localized closely to the cortex or in the limbic or perilimbic areas are highly epileptogenic, whereas tumors located in the inner parts of the brain are less prone to manifest seizures (Berntsson et al., 2009; Cowie and Cunningham, 2014). The pathogenesis of TAE is likely to be multifactorial and involves the interaction of genetic factors, changes in the peritumoral microenvironment, hypoxia, acidosis, and metabolic impairments that could affect neural morphology and function (You G. et al., 2012; Cowie and Cunningham, 2014; Armstrong et al., 2016). In addition, the balance between excitatory and inhibitory networks certainly plays a pivotal role in the generation of seizures (Nelson and Turrigiano, 1998; You Y. et al., 2012; MacKenzie et al., 2017). Indeed, the high amount of glutamate extruded by glioma cells overactivates both NMDA and AMPA receptors resulting in excitotoxicity, tumor invasion and hyperexcitability of neural tissues (Rzeski et al., 2001; Savaskan et al., 2008; Sontheimer, 2008; Vanhoutte and Hermans, 2008; Marcus et al., 2010; Robert and Sontheimer, 2014). The prolonged activation of NMDA receptors provokes a sustained influx of Ca^2+^ into the cell mediating excitotoxicity (Terunuma et al., 2010), whereas the reduction of neuronal cell density creates room for tumor expansion (Buckingham et al., 2011) and the overactivation of AMPA receptors further boosts neural excitability (Savaskan et al., 2008). In order to adapt to a glutamate-rich environment and avoid the toxic effects of glutamate, glioma cells downregulate AMPA or NMDA receptors in a grade- and spatial-dependent manner. Indeed, expression analyses of different tumor regions showed that their expression is highest at the infiltrating front of the tumors, suggesting a role in mediating tumor invasion (Radin and Tsirka, 2020).

Multiple factors are necessary to establish long-lasting hyperexcitation and concur to support the onset of peritumoral epilepsy (Campbell et al., 2015). For instance, pyramidal peritumoral neurons downregulate KCC2 and upregulate NKCC1, two transporters responsible for exporting and importing chloride, respectively. The consequent abnormal high amount of intracellular chloride causes GABA-mediated depolarization, reversing chloride potential and leading to an excitatory and detrimental action of GABA receptor in pyramidal neurons (Conti et al., 2011; Di Angelantonio et al., 2014; Pallud et al., 2014; Campbell et al., 2015; Huberfeld and Vecht, 2016; Jung et al., 2019). In addition, peritumoral regions show a significant loss of approximately 35% of GABAergic interneurons, together with a reduction in their firing rates and in synapses with pyramidal neurons (Campbell et al., 2015; Tewari et al., 2018; Yu et al., 2020). Both altered chloride homeostasis and decreased GABAergic inhibition contribute to render the peritumoral tissue more prone to seizures (Buckingham et al., 2011; Venkatesh et al., 2019).

Finally, several studies have shown that the perineuronal nets (PNNs), which surround fast spiking interneurons acting as ionic buffer, neuroprotecting and stabilizing their synaptic activity, resulted degraded by glioma-released proteases. This provokes an increased membrane capacitance and, in turn, reduces the firing rate of the remaining peritumoral inhibitory interneurons within 400 μm from tumor edge. In particular, the pro-seizure effect of PNN degradation seems to be due to the lacking role of electrostatic insulators affecting the physiological properties of fast-spiking interneurons (Buckingham et al., 2011; Tewari et al., 2018; Venkatesh et al., 2019).

Astrocytomas are able to directly interact with the surrounding environment, extending ultra-long membrane protrusions called tumor microtubes (TMs) that represent a route for brain invasion (Osswald et al., 2015). Recently, it has been discovered that glioma cells can create an intricate interconnection with neurons, forming an electrically coupled network. Within these networks neuronal activity evokes non-synaptic activity-dependent potassium currents amplified by tumor mediated gap junctions (Venkataramani et al., 2019, 2021). The presence of these structures correlates with the worst prognosis in human gliomas and confers resistance against all the available therapies (Osswald et al., 2015; Weil et al., 2017). Studies in Drosophila demonstrate that brain tumor cells protrude TMs to enwrap neurons, inhibiting the Wnt pathway through the accumulation of Fz1 receptor or through the secretion of pathway antagonists (such as Impl2), thus causing neurodegeneration (Portela et al., 2019; Jarabo et al., 2021). In addition, glioma cells interact with peritumoral excitatory neurons forming functional bona fide AMPA-mediated synapses (Venkataramani et al., 2019), through which pro-mitotic peptides as NLGN3 can stimulate glioma progression (Venkatesh et al., 2019). Surprisingly, even tumor cells of non-brain origin, like metastatic breast cancer cells, establish synaptic contacts with neurons (Zeng et al., 2019). This suggests that tumor-neuron synapses are an efficient way that tumor cells exploit in order to obtain glutamate and sustain their proliferation.

## Neuronal Alterations Induced by Glial Cells in the TME

In addition to neuronal alterations directly induced by glioma cells, glial cells response can also profoundly affect brain functionality. Being coopted by tumor cells, glial cells can no longer support brain homeostasis. In addition, activated glial cells contribute to establish a TME milieu rich of cytokines, metabolites and signaling molecules that have potential detrimental effects on neuronal activity. The inflammatory status of gliomas is the result of complex interactions of different TME players. Astrocytes, TAMs and oligodendrocytes all possess immunomodulatory properties, including the production of a broad spectrum of cytokines. Beside their prominent role in stimulating or suppressing immune and glial cells activity, the cytokines present in the TME can also affect neuron survival and activity. Although there are some examples of chemokines with neuroprotective roles (Trettel et al., 2020), an excessive accumulation of pro- and anti-inflammatory cytokines has usually neurotoxic effects. Cytokines like IL1α/β, TNFα, and TGFβ, which are highly enriched within the TME, have been shown to induce neurons loss, impair neuronal morphogenesis and are associated with many neurodegenerative diseases (Glass et al., 2010; Nakashima et al., 2018).

Inflammation is also a consequence of aggressive chemo- and radiotherapies that can exacerbate a neurotoxic microglia-astrocytes crosstalk with long-term consequences on neurons integrity, myelin plasticity as well as neurogenesis (Monje et al., 2003; Gibson et al., 2019). It is well-known that anti-cancer treatments, beyond their unquestionable advantage of delaying tumor progression, are accompanied by multiple side effects that in most of the cases are short-term and temporary. However, what is less known are the long-term consequences of these therapies, especially on brain functions, with patients experiencing deficits in memory, attention, information process, and even mental health (Gibson and Monje, 2021). In addition, neurotoxicity associated with cerebral edema has been reported after immunomodulatory treatments, although the underlying mechanisms are still undefined (Gust et al., 2017; Spain et al., 2017).

Cerebral edema is a common complication of brain tumors and is generally associated with increased intracranial pressure, reduced blood flow and hypoxia, all events that lead to neuronal deficits (Roth et al., 2013). These phenomena can arise as consequence of the presence of a tumor mass, that physically compresses and remodels the brain parenchyma. For example, by migrating along blood vessels, tumor cells displace astrocytic endfeet from endothelial cells causing loss of endothelial tight junctions and breaches in the BBB (Watkins et al., 2014). Similar effects have also been described in some multiple sclerosis active plaques, where OPCs, due to a defective migration, accumulate along the blood vessels disrupting the BBB. Mechanistically, an excessive Wnt signaling in OPCs triggers perivascular accumulation and release of Wif-1 which inhibits Wnt ligand essential for endothelial cells tight junction, compromising the integrity of the endothelial barrier (Niu et al., 2019). OPCs expressing Wif-1 were also found in gliomas (Asslaber et al., 2017), suggesting that their influence on BBB homeostasis might not be limited to multiple sclerosis but might be extended to brain tumors. Disruption of the neurovascular coupling during disease progression impairs the hemodynamic response after seizures, causing hypoxia and exacerbating the seizure-related brain damages (Montgomery et al., 2020). Glial and immune cells in the TME can also actively contribute to cerebral edema. Indeed, BBB and vessels permeability are altered by cytokines and factors, like VEGF, released by tumor cells, astrocytes and TAMs (Calabrese et al., 2007; Priego et al., 2018; Sankowski et al., 2019; Klemm et al., 2020). Beside its pro-angiogenic functions, VEGF can induce expression of AQ4, a water-channel that is mainly present on astrocytes composing the BBB and that controls its permeability (Lan et al., 2017). Anti-angiogenic treatments that normalized tumor vasculature have demonstrated efficacy also in reducing brain edema in GBM patients (Batchelor et al., 2007). Corticosteroids are the drug of choice to treat glioma-induced cerebral edema for their ability to downregulate VEGF, modulate the expression of channels and tight junctions’ components and, therefore, limiting BBB permeability. However, among other side effects, corticosteroids, and in particular dexamethasone, show immunosuppression and might have additional and unknown effects on cells of the TME that deserve further scrutiny (Cenciarini et al., 2019).

As discussed earlier, PNNs degradation alters neuronal activity concurring to TAE. The contribution of glial cells to PNNs remodeling has been widely demonstrated in physiological conditions and in other diseases (Crapser et al., 2020; Nguyen et al., 2020), but it remains speculative in glioma. Indeed, TAMs, astrocytes and OPCs are important sources of MMPs and can assist tumor cells in ECM remodeling, possibly including PNNs degradation. Exposure to an EE, achieved by continuous physical, sensory and social stimulation, is also known to shape ECM and PNNs, to induce plasticity and neurogenesis and to modulate microglia and astrocytes activation (Baroncelli et al., 2010; Rodríguez et al., 2013; Kempermann, 2019; Yates, 2020). Interestingly, an EE is sufficient to significantly prolong the survival of glioma-bearing mice through re-education of TAMs toward a less immunosuppressive state and through IL15-mediated recruitment of anti-tumor NK cells, which also contribute to TAMs re-polarization (Garofalo et al., 2015, 2017).

Neuron hyperexcitability and acute seizures could be attenuated by microglia *via* P2RY12 (Eyo et al., 2014) which is, however, downregulated in glioma (Sankowski et al., 2019; Friebel et al., 2020; Guldner et al., 2020; Klemm et al., 2020). In a zebrafish brain tumor model, this mechanism was hijacked by tumor cells that transiently increased intracellular Ca^2+^ levels and ATP release, in order to coopt P2RY12^+^ microglia and establish direct microglia-to-tumor contacts that stimulate tumor proliferation (Chia et al., 2019). It remains to be investigated whether tumor cells can exploit also the synapse pruning ability of microglia and astrocytes to form and remodel glioma synapses, possibly at the expense of neurons (Paolicelli et al., 2011; Chung et al., 2013). Of note, specific astrocyte subpopulations have been shown to support synapse formation and their emergence in gliomas correlates with increased hyperexcitability and seizures (John Lin et al., 2017).

In the healthy brain, astrocytes are responsible for removing the excess of extracellular glutamate *via* EAAT1/2 transporters and converting glutamate into glutamine, which is released and re-used by neurons (Mahmoud et al., 2019). However, in gliomas, peritumoral reactive astrocytes show altered electrophysiological properties as well as decreased glutamate uptake and glutamine production, that lead to GABAergic disinhibition and neuronal hyperexcitability (Campbell et al., 2020). Therefore, reactive astrocytes might propagate the glutamate excitotoxicity and exacerbate, instead of preventing, neuronal death and glioma-induced epilepsy.

## Conclusion and Future Perspectives

**FIGURE 1:**
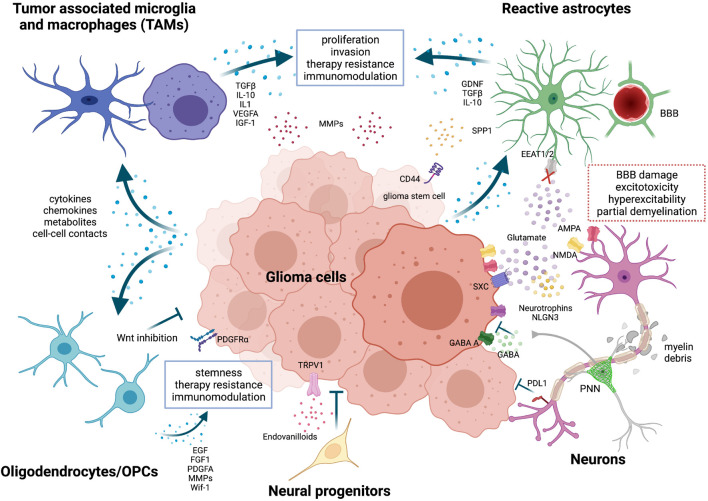
Neurons and glial cells in the glioma microenvironment. Tumor-associated microglia and macrophages (TAMs) are reprogrammed by tumor cells through the release of cytokines, chemokines, metabolites or through cell-cell contacts. Activated TAM produce factors that stimulate glioma proliferation, invasion, angiogenesis, and therapy resistance. Metalloproteinases (MMPs) are released by both tumor cells and TAMs to facilitate glioma cells migration. TME-associated astrocytes are efficiently coopted by tumor cells. They promote tumor migration by expressing chemoattractant factors like GDNF and support stemness *via* SPP1-CD44 axis. Both TAMs and astrocytes also play a role in immunomodulation, expressing high levels of immunosuppressive cytokines, such as TGFβ and IL-10. Oligodendrocytes and oligodendrocyte progenitor cells (OPCs) release factors like EGF, FGF1 and PDGFA that favor proliferation, stemness and chemotherapy resistance of glioma cells. But, at the same time, OPCs can also impair tumor growth through Wnt inhibition. Neural progenitor cells delay tumor growth and induce tumor cell death *via* endovanilloids stimulation of TRPV1. Neurons have a multifaceted role in glioma. Peritumoral neurons were found to counteract glioma progression through programmed death-ligand 1 (PD-L1) protein, but recent evidence has shown that the activation of peritumoral excitatory neurons was able to increase glioma proliferation. Selective activation of peritumoral parvalbumin interneurons elicited, instead, the opposite effect. In addition, tumor cells extrude glutamate outside predominantly through the overexpression of the cystine-glutamate antiporter (system xc-, SXC); this event is not harmonized by peritumoral astrocytes, which show a compromised glutamate uptake *via* the Na^+^-dependent glutamate transporters (i.e., EAAT1 or EAAT2). High amount of glutamate provokes an over-activation of both NMDA and AMPA receptors on neuronal cells, resulting in excitotoxicity and hyperexcitability of neural tissues and eventually promotes survival, growth and migration of glioma cells. Glutamatergic neurons can enhance glioma progression also by releasing NLGN3 and neurotrophins and by establishing functional bona fide AMPA-mediated synapses with tumor cells. Invading glioma cells, together with activated glial cells in the tumor microenvironment, cause also secondary alterations like perineural nets (PNNs) remodeling, partial demyelination and blood-brain-barrier (BBB) leakage. Image created with BioRender.com.

In physiological conditions a balance among glial cells, immune cells and neurons is necessary and established with the final goal of sustaining neuronal activity and brain homeostasis. In case of malignancy, tumor cells represent an additional player introduced in the game, forcing the equilibrium at its advantage. Indeed, tumor cells coopt neurons and glial cells to create a microenvironment that influences their growth (Figure 1). In this context, new interactions are established between tumor and brain resident cells. Interestingly, some of these interactions vary depending on the type of glioma, suggesting a preferred mode of communication that might be intrinsically related to the tumor cell-of-origin or be dictated by specific mutations (Gao et al., 2020).

Tumor cells exploit and even strengthen pre-existing interactions within the TME. Astrocytes, TAMs and OPCs/oligodendrocytes actively communicate and modulate each other’s activation in physiological conditions, as well as during tumor progression. Yet, neuronal activity is affected by direct interaction with tumor cells and also indirectly by changes in the TME. Although some of the cellular and molecular mechanisms behind these complex networks have been recently uncovered, many aspects still need to be further investigated. For example, it still remains unclear to what extent cell-cell interactions dynamically change during disease progression and in response to treatments. In addition, cell types like OPCs, that have been so far neglected in gliomas, definitely deserve more attention.

Novel therapeutic strategies that target the TME have recently emerged, showing promising results especially when used in combination with other treatments (Kowal et al., 2019). This suggests that acting on several fronts is a successful strategy to impair cellular interplays in the TME and tumor-TME crosstalk. Nevertheless, there is still a great deal to be done. Indeed, in glioma patients, clinical trials of TME-targeting therapies, and especially immunomodulatory therapies, didn’t meet the expectations. Part of the failure is certainly due to the intrinsic therapeutic resistance of gliomas and the particularly immunosuppressed brain microenvironment (Sampson et al., 2020). However, it would be also important to understand the contribution of cells in the TME that are not directly targeted by these treatments.

Interestingly, cells in the TME can undergo senescence due to aging (Fane and Weeraratna, 2019) or upon chemo/radiotherapy (Wang et al., 2020). Cellular senescence is a complex cellular state which determines not only cell intrinsic effects, like cycle arrest and DNA damage response, but also non-cell-autonomous mechanisms that influence the surrounding microenvironment. The senescence-associated secretory phenotype (SASP) involves the secretion of pro-inflammatory factors, including cytokines and MMPs, that promote various aspects of tumor development like tumor cell proliferation (Kuilman et al., 2008) and immunosuppression (Ruhland et al., 2016). Although numerous studies have reported that non-malignant cells can acquire a senescent phenotype and promote tumor progression in multiple cancers, little is known about senescent non-malignant cells in the brain TME (Wang et al., 2020). Chemotherapy triggers in microglia and astrocytes a pro-inflammatory secretome (Gibson et al., 2019), which is similarly induced by physiological aging (Li et al., 2015; Clarke et al., 2018) or neurodegenerative diseases (Bhat et al., 2012; Liddelow et al., 2017). Progenitor cells and OPCs also show a senescence phenotype in pathological conditions (Nicaise et al., 2019; Zhang et al., 2019) and secondary detrimental effects of senescent glial cells have been reported also on neurons (Limbad et al., 2020). Senolytic therapies have been efficiently used to eliminate senescent cells in multiple diseases, including cancers (Sieben et al., 2018). JAK2/STAT3 inhibitors, beside their immunomodulatory role on tumor-associated astrocytes (Priego et al., 2018), have been used to reprogram SASP and delay prostate cancer growth (Toso et al., 2014). Moreover, CAR T cells, genetically modified T cells which usually recognize tumor cells-specific antigens, have also been recently engineered to target senescent cells, with promising results in mouse models (Amor et al., 2020). Interestingly, the senescence-specific antigen used to target senescent cells was uPAR, which is also highly upregulated by tumor-associated astrocytes (Le et al., 2003). Therefore, senolytic treatments could be considered as an alternative strategy to target the TME and might be used in combination with more classic anticancer therapies. However, further investigations are certainly necessary to unravel the mechanisms inducing senescence in brain TME cells and in order to identify the factors produced by senescent cells to sustain tumor progression.

We should also not forget secondary alterations, that can be due to direct detrimental effects of tumor cells or to indirect glial cells dysregulation in the TME. In both cases such alterations profoundly affect brain functionality and, consequently, patients’ quality of life; therefore, these events should be considered in the choice of the therapeutic intervention and in the development of alternative treatments.

Finally, some technical considerations should be made. We have here reviewed the most important recent studies that have contributed to add pieces to the complex jigsaw of the brain TME, employing different models (e.g., human, mouse, Drosophila, zebrafish) and approaches (e.g., *in vitro*, *in vivo*). Certainly, every model has its own advantages and disadvantages that must be taken into account in the interpretation of the obtained results; remarkably, using different approaches could help to tackle the issue from different points of view, producing a more complete and reliable scenario of the cellular and molecular players in the TME. Since the immune system exerts a critical role in the TME (Lei et al., 2020; Sampson et al., 2020) and, as reported in this review and in many studies, all the TME cells possess immunomodulatory functions, it would be desirable to choose models that do not exclude this important player of the TME (i.e., immunocompetent animal models). In order to address some of the current gaps of knowledge, innovative experimental approaches have been more recently developed. Patients-derived organoids or organotypic cultures are, for example, 3D *in vitro* models that reproduce the TME interactions and could be very useful for testing novel drugs or screening the patient-specific response and subsequently choose the proper therapeutic intervention (Pamies et al., 2020). However, due to their complexity, at the moment these types of models are of difficult adaptation to high-throughput screening. New technologies are also currently aiming at overcoming other limitations, such as the integration of microfluidics or 3D bioprinting of tissue structures to circumvent the lack of a complete vasculature system and the infiltration of peripheral immune components (Li et al., 2020; Pamies et al., 2020).

Dissecting the contribution of different cells of the TME and understanding their interactions is of paramount importance to shed light on the mechanisms that glioma cells exploit to support their growth and invasion. A profound knowledge of the TME is a puzzling but relevant issue, because it will highlight the molecules that could be targeted for the development of novel therapeutic strategies aimed at counteracting brain tumors.

## Author Contributions

EP, ET, and EV: conceptualization. EP, MS, and EV: review and editing. EP, MS, EM, ET, and EV: contribution to draft preparation. MS and EM: figure making. All authors contributed to the draft preparation, have read and agreed to the published version of the manuscript.

## Conflict of Interest

The authors declare that the research was conducted in the absence of any commercial or financial relationships that could be construed as a potential conflict of interest.

## Publisher’s Note

All claims expressed in this article are solely those of the authors and do not necessarily represent those of their affiliated organizations, or those of the publisher, the editors and the reviewers. Any product that may be evaluated in this article, or claim that may be made by its manufacturer, is not guaranteed or endorsed by the publisher.

## Glossary

**Table:**

ADAM10	A Disintegrin And Metalloprotease domain 10
AKT	Serine/Threonine Kinase
AKT1	Serine/Threonine Kinase 1
AMPA	Alpha-amino-3-hydroxy-5-Methyl-4-isoxazolePropionic Acid
AMPAR	Alpha-amino-3-hydroxy-5-Methyl-4-isoxazolePropionic Acid Receptor
AQ4	Aquaporin-4 ATP Adenosine Triphosphate BBB Blood Brain Barrier
BDNF	Brain-Derived Neurotrophic Factor
CAR T	Chimeric Antigen Receptor
T CCL2	C-c motif Chemokine Ligand 2
CCL5	C-c motif Chemokine Ligand 5
CCR2	C-c motif Chemokine Receptor 2
CD11b	Cluster of Differentiation molecule 11b
CD11c	Cluster of Differentiation molecule 11c
CD133	Cluster of Differentiation molecule 133
CD169	Cluster of Differentiation molecule 169
CD206	Cluster of Differentiation molecule 206
CD209	Cluster of Differentiation molecule 209
CD44	Cluster of Differentiation molecule 44
CD45	Cluster of Differentiation molecule 45
CD47	Cluster of Differentiation molecule 47
CD49d	Cluster of Differentiation molecule 49d
CNS	Central Nervous System
CSF-1R	Colony Stimulating Factor 1 Receptor
CSF1	Colony Stimulating Factor 1
CX3CL1	C-X3-c motif Chemokine Ligand 1
Cx43	Connexin 43
Cxcr4	C-x-c motif chemokine receptor 4
EAAT1	Excitatory Amino Acid Transporter 1
EAAT2	Excitatory Amino Acid Transporter 2
ECM	Extracellular Matrix
EE	Enriched Environment
EGF	Epidermal Growth Factor
EGFR	Epidermal Growth Factor Receptor
FasL	Fas Ligand
FGF1	Fibroblast Growth Factor 1
Fz1	Frizzled class receptor 1
GABA	Gamma-Aminobutyric Acid
GABA A	Gamma-Aminobutyric Acid type A
GABA B	Gamma-Aminobutyric Acid type B
GBM	Glioblastoma Multiforme
GDNF	Glial cell line-Derived Neurotrophic Factor
GFAP	Glial Fibrillary Acidic Protein
GLAST	Glutamate-ASpartate Transporter
GLUD2	Glutamate Dehydrogenase 2
Hexb	Hexosaminidase subunit beta
HIF1A	Hypoxia Inducible Factor 1 subunit Alpha
Iba1	Ionized calcium-binding adapter molecule 1
IDH	Isocitrate Dehydrogenase
IFNγ	InterFeroN Gamma
IGF-1	Insuline-like Growth Factor 1
IL1	Interleukin 1
IL6	Interleukin 6
IL10	Interleukin 10
IL15	Interleukin 15
IL1α	Interleukin 1 alpha
IL1β	Interleukin 1 beta
Impl2	Imaginal morphogenesis protein-late 2
Ink	Inhibitors of cyclin-dependent kinase
Ink4A/Arf or p16	Inhibitors of cyclin-dependent kinase 4a/Alternate reading frame
JAK/STAT	Janus Kinase/Signal Transducers and Activators of Transcription
KCC2	Potassium Chloride Cotransporter 2
Lgals3	Galectin 3
MARCO	Macrophage Receptor with Collagenous structure
MDMs	Monocyte-Derived Macrophage
MerTK	Mer Tyrosine Kinase
MHC-I	Major Histocompatibility Complex class I
MHC-II	Major Histocompatibility Complex class II
MMP2	Matrix MetalloPeptidase 2
MMP9	Matrix MetalloPeptidase 9
MT1-MMP	Membrane Type 1 Matrix MetalloProteinase
NF1	NeuroFibromatosis type 1
NG2	Neural/Glial antigen 2
NGF	Nerve Growth Factor
NK	Natural Killer
NKCC1	Na-K-Cl Cotransporter 1
NLGN3	Neuroligin 3
NMDA	N-Methyl-D-Aspartic acid or N-Methyl-D-Aspartate
NMDAR	N-Methyl-D-Aspartic acid or N-Methyl-D-Aspartate Receptor
NSC	Neural Stem Cell
OPC	Oligodendrocyte Progenitor Cell
P2RY12	Purinergic Receptor P2Y12
PA	Plasminogen Activator
PD-1	Programmed cell Death protein 1
PD-L1	Programmed Death-Ligand 1
PDGFA	Platelet-Derived Growth Factor subunit A
PDGFB	Platelet-Derived Growth Factor subunit B
PDGFC	Platelet-Derived Growth Factor C
PDGFRA/PDGFRα	Platelet-Derived Growth Factor Receptor Alpha
PDGFRβ	Platelet-Derived Growth Factor Receptor beta
PI3K	Phosphoinositide 3-Kinase
PNNs	Perineuronal Nets
PTEN	Phosphatase and Tensin homologue
RhoA	Ras homolog family member A
Sall1	Spalt like transcription factor 1
SASP	Senescence-Associated Secretory Phenotype
Sema3A	Semaphorin 3A
SHH	Sonic HedgeHog signaling molecule
SOX10	Sry-box transcription factor 10
SOX2	Sry-box transcription factor 2
SOX9	Sry-box transcription factor 9
SPP1	Secreted Phosphoprotein 1
STAT3	Signal Transducer and Activator of Transcription 3
SXC	System XC- cystine/glutamate antiporter
TAE	Tumor-Associated Epilepsy
TAMs	Tumor-Associated Microglia/Macrophages
TGFβ	Transforming Growth Factor Beta
TLR3	Toll-Like Receptor 3
TME	Tumor Microenvironment
Tmem119	Transmembrane protein 119
TMs	Tumor Microtubes
TNFα	Tumor Necrosis Factor Alpha
TrkA	Tropomyosin receptor kinase A
TrkB	Tropomyosin receptor kinase B
TrkB.T1	Truncated isoform of tropomyosin receptor kinase B
TRPV1	Transient Receptor Potential Vanilloid 1
uPA	urokinase-type Plasminogen Activator
uPAR	urokinase-type Plasminogen Activator Receptor
VEGF	Vascular Endothelial Growth Factor
VEGF-A	Vascular Endothelial Growth Factor A
Wif-1	Wnt inhibitory factor 1
Wnt	Wingless/integrated.
